# Abdominal Malignancy with Skin Manifestation and Thromboembolic Disease: An Unusual Presentation

**DOI:** 10.7759/cureus.7302

**Published:** 2020-03-18

**Authors:** Raguraj Chandradevan, Jennifer Espinal, Marika Shahid, Randolph Devereaux, Tarun K Ghosh

**Affiliations:** 1 Internal Medicine, Coliseum Medical Centers, Macon, USA; 2 Preventive Medicine, Coliseum Medical Centers, Macon, USA

**Keywords:** sister mary joseph’s nodule, thromboembolism, gastrointestinal malignancy

## Abstract

A 55-year-old male presented to our hospital with shortness of breath and leg swelling. Imaging studies revealed deep vein thrombosis and bilateral pulmonary embolism. The patient was placed on anticoagulation. A palpable umbilical nodule consistent with the appearance of Sister Mary Joseph's nodule (SMJN) raised the possibility of an underlying gastrointestinal malignancy. The patient also had significant ascites and underwent paracentesis with cytology, upper gastrointestinal and lower gastrointestinal endoscopy with the biopsy. Gastric lesion histology revealed gastric adenocarcinoma with peritoneal and colonic metastases. The patient was started on chemotherapy with 5‐fluorouracil, leucovorin, oxaliplatin (FOLFOX) for disseminated gastric malignancy. SMJN is a rare cutaneous metastatic manifestation which needs to be considered as a differential diagnosis of an umbilical tumor for prompt diagnosis and initiation of treatment.

## Introduction

Sister Mary Joseph's nodule (SMJN) is a rare and peculiar physical sign found in 1%-3% of patients with an intrabdominal or pelvic malignancy [1]. The physical finding is named after Sister Mary Joseph, a surgical assistant to Dr. William J. Mayo, who noted the association between the presence of the nodule and an underlying malignancy [2]. It is a morphological representation of cutaneous metastases with an underlying intra-abdominal tumor with an extensive invasion through the peritoneal cavity. The mechanism of spread of the tumor to the umbilicus is poorly understood as it seems to be lymphatic, vascular, contiguous, or via embryological remnants in the abdominal wall [3]. Commonly encountered primary tumors are gastrointestinal malignancies including gastric, colonic, and pancreatic which account for 52% of cases. Gynecological cancers account for about 28% and the rest of the cases have an unknown origin [4]. The likelihood of SMJN presenting as an initial clinical feature and associated venous thromboembolism during the first encounter is extremely rare in the literature. Here, we present a case of advanced gastric adenocarcinoma, its diagnosis, treatment, and follow-up. 

## Case presentation

A 55-year-old African American male with a past medical history of hypertension presented to the emergency department at our hospital with a chief complaint of generalized weakness. He experienced an acute onset of shortness of breath that started a couple of days prior to his presentation. He was working in his back yard and suddenly became dizzy and felt like he could not catch his breath. He attributed this episode to an anxiety attack due to ongoing stress factors. He endorsed some level of stress due to a bout of persistent diarrhea he had been experiencing for one month. The diarrhea started off with a few soft bowel movements per day and increased to up to six or seven bowel movements daily. The bowel movements were watery, without blood or mucus. These episodes of diarrhea were associated with mild, dull abdominal aches, and progressive abdominal distension. He also reported a 40-pound weight loss in the past year which he attributed to dietary changes that he had made for his gastroesophageal reflux disease (GERD). He also endorsed a decreased appetite for four weeks. In addition, two weeks ago, he noticed swelling in his right calf and thigh, followed by the swelling of his left leg.

**Figure 1:**
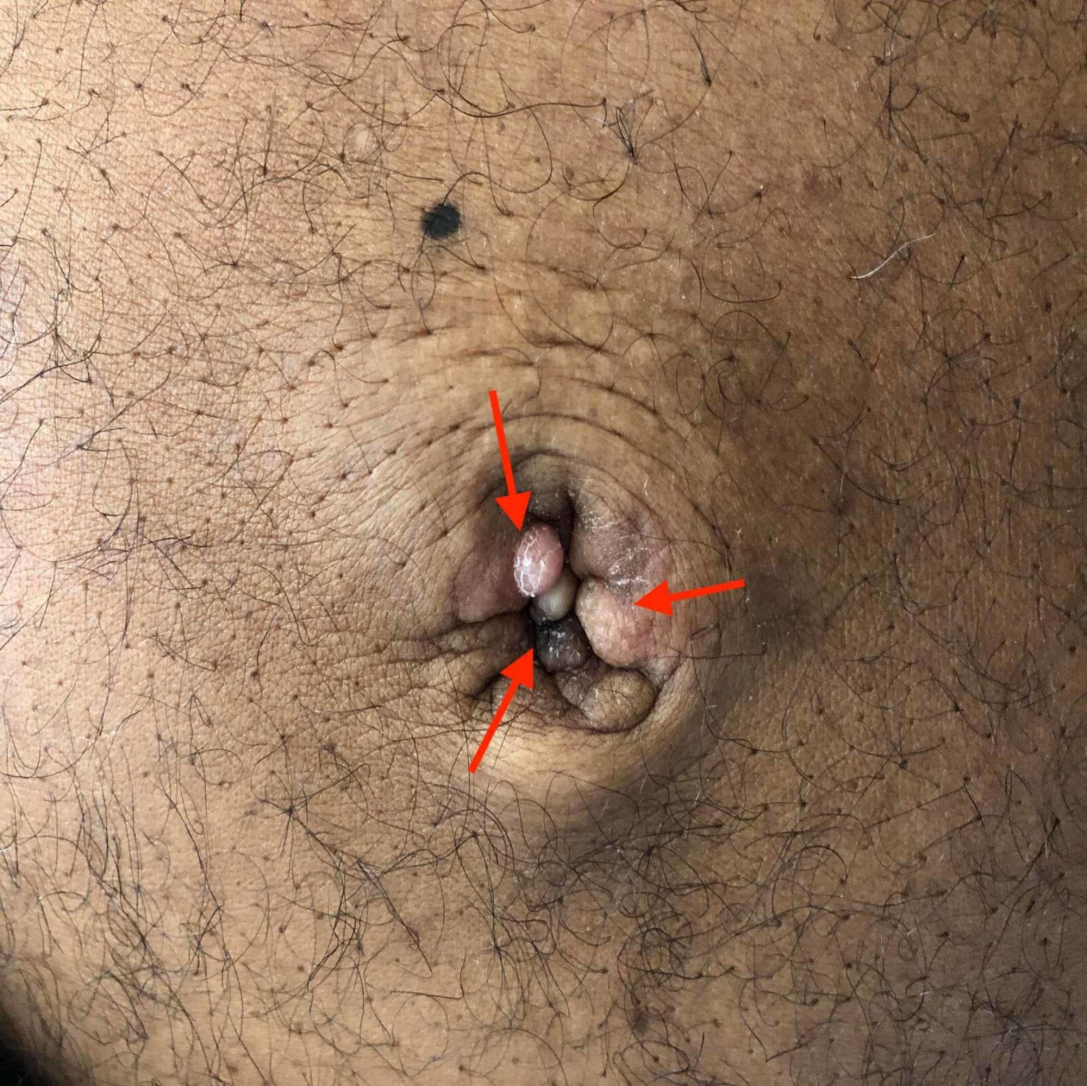
Irregular and pink nodules (red arrows) at the umbilicus

He was hypotensive on arrival at the emergency department with low blood pressure (BP) and a systolic blood pressure (SBP) in the 80s and diastolic blood pressure (DBP) in the 50s (mmHg) and tachycardic with a heart rate of 110 beats per minute (bpm). He was alert and oriented times four. Pertinent physical examination findings were of lower extremity swelling up to the mid-thigh level (pitting). Distended, non-tender abdomen with fluid thrills were suggestive of ascites. Further abdominal exam revealed a 5 x 4 cm palpable umbilical nodule which was purplish, non-tender and suggestive of possible SMJN (Figure 1). He had clear breath sounds bilaterally and had +2 pitting edema in the left lower leg.

**Figure 2:**
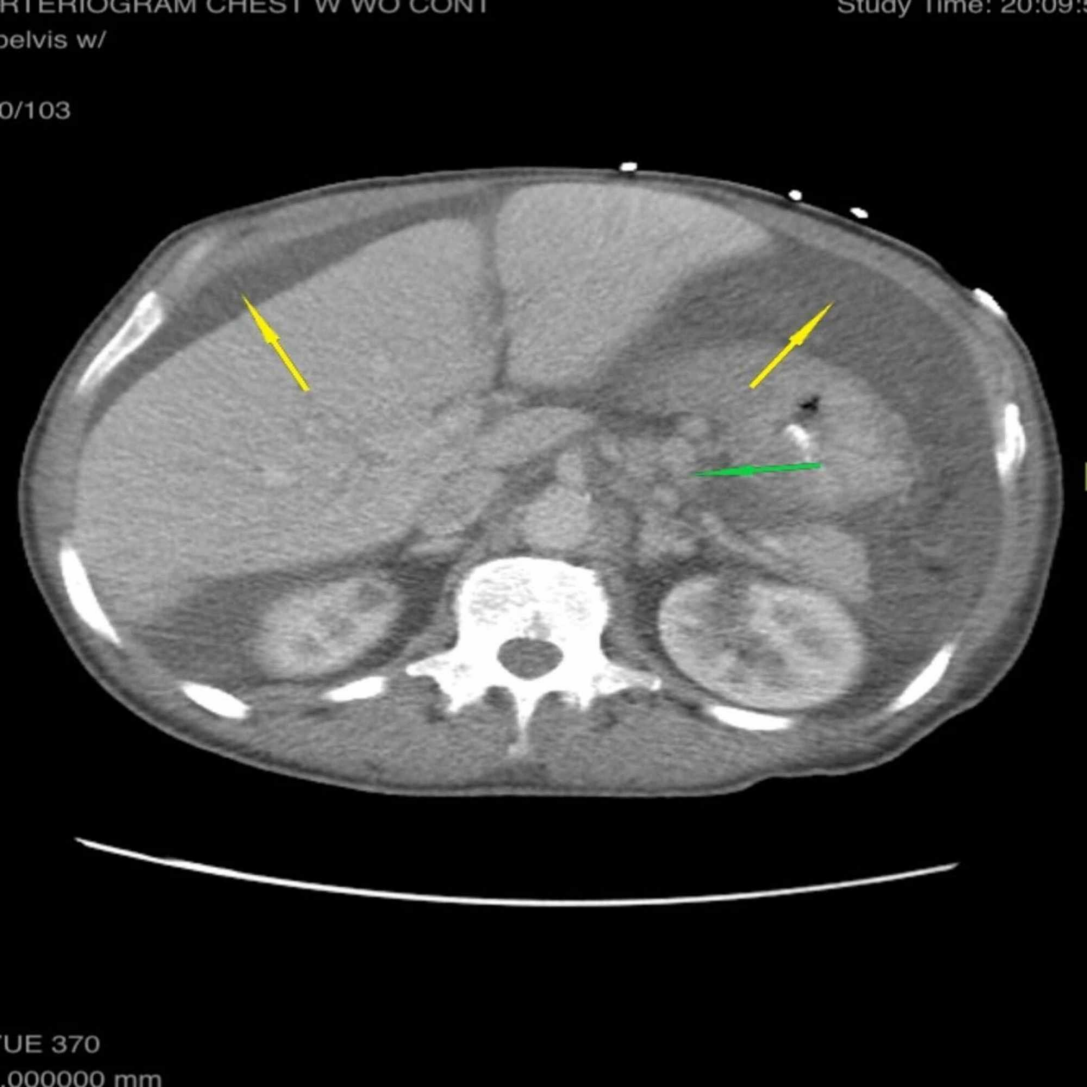
Non-contrast computed tomography of the abdomen in axial view showing gastric fundal varices (green arrow) and large volume ascites (yellow arrows)

**Figure 3:**
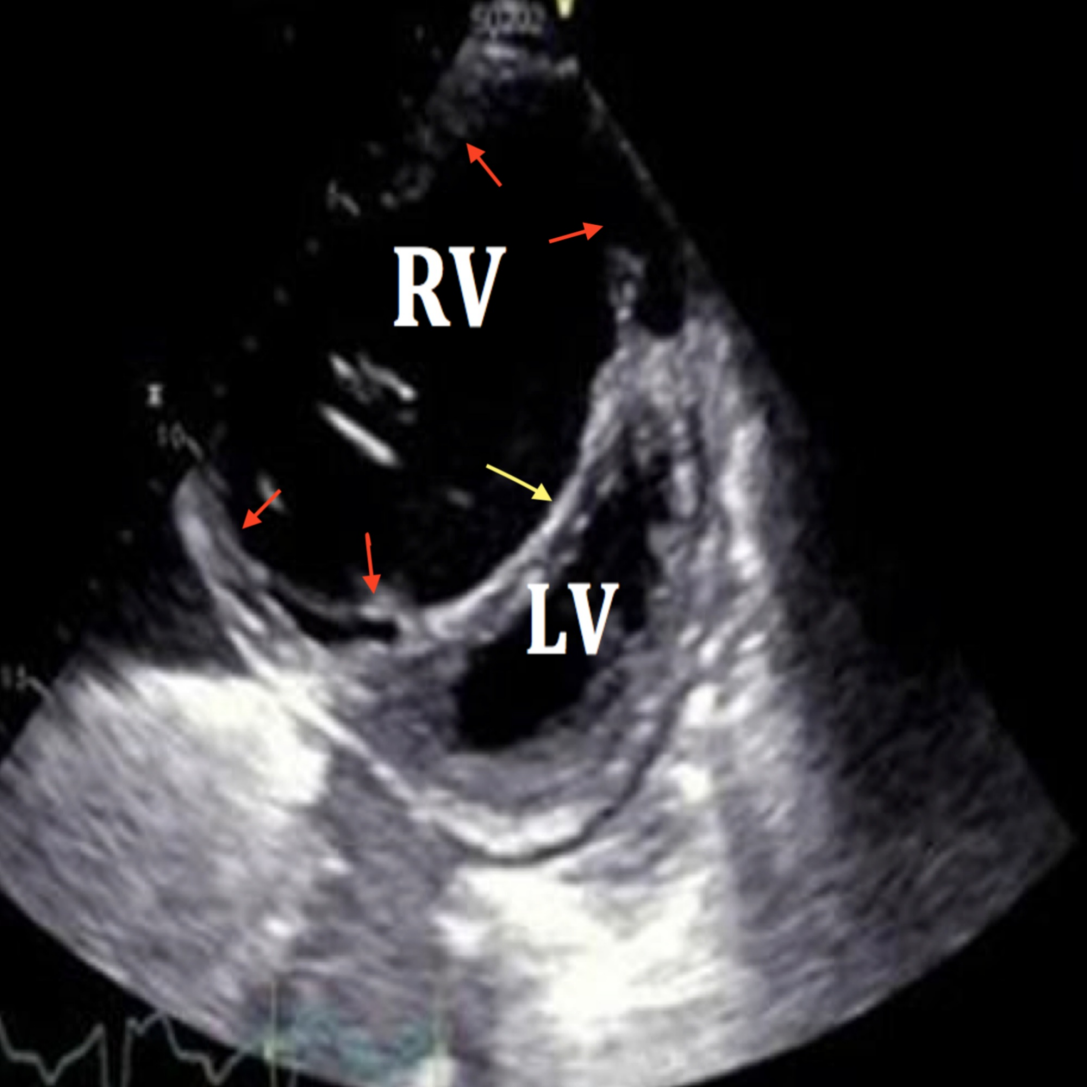
Parasternal short axis view of right ventricular (RV) dilatation (red arrows) and inter-ventricular (LV) septal shift (yellow arrow)

His Wells score for pulmonary embolism (PE) was 7.5 putting him at high risk of PE. He had computed tomography angiography (CTA) of the chest, Doppler ultrasound scan of the bilateral lower extremities (venous) and an urgent 2D transthoracic echocardiography (2D TTE) due to his hemodynamic instability. Upon further history taking, he denied any recent travel, trauma, surgery, and any family history of thrombophilia or other blood disorders. Our initial lab work revealed white blood cells (WBC) of 17.000, haemoglobin (Hgb) of 12.5 mg/dL, normal electrolytes except for hypokalemia with a potassium level of 2.8 mEq/L. His troponin-I was high with an initial value of 0.166 ng/mL, and D-dimer level was 7 mg/L. His prothrombin time (PT) and partial thromboplastin time (PTT) were normal. His lactate dehydrogenase (LDH) was elevated to 215. CTA of the chest revealed bilateral lobar, segmental and sub segmental pulmonary thromboembolism (PTE) some of which were occlusive, and numerous others were predominantly non-occlusive with patent pulmonary artery. There was straightening of the interventricular septum and bowing into the left ventricle which suggested a pattern of right heart strain. Further extension of the computed tomography (CT) to the abdomen and pelvis revealed large volume ascites, diffuse thickening of the stomach, small bowel, and colon likely secondary to edema. Gastroesophageal varices were also seen (Figure 2). The 2D echocardiogram findings showed a normal left ventricular function and a markedly dilated right atrium and right ventricle with inter-ventricular septal shift (Figure 3). Pulmonary artery systolic pressure was increased and estimated peak pressure was at least 50 mmHg.

On further evaluation, the patient remained asymptomatic. His oxygen saturation remained normal. He was hemodynamically stabilized with volume resuscitation. Initially, thrombolytic option was considered, but was ultimately deferred since he had esophageal varices on CT in addition to a positive fecal occult blood test. He was started on high-dose heparin (weight based protocol). Doppler ultrasound scan of bilateral extremities revealed occlusive deep vein thrombosis in his left superficial femoral vein, popliteal, and posterior tibial veins. Once stabilized, further work up for the primary source was initiated. 

**Figure 4:**
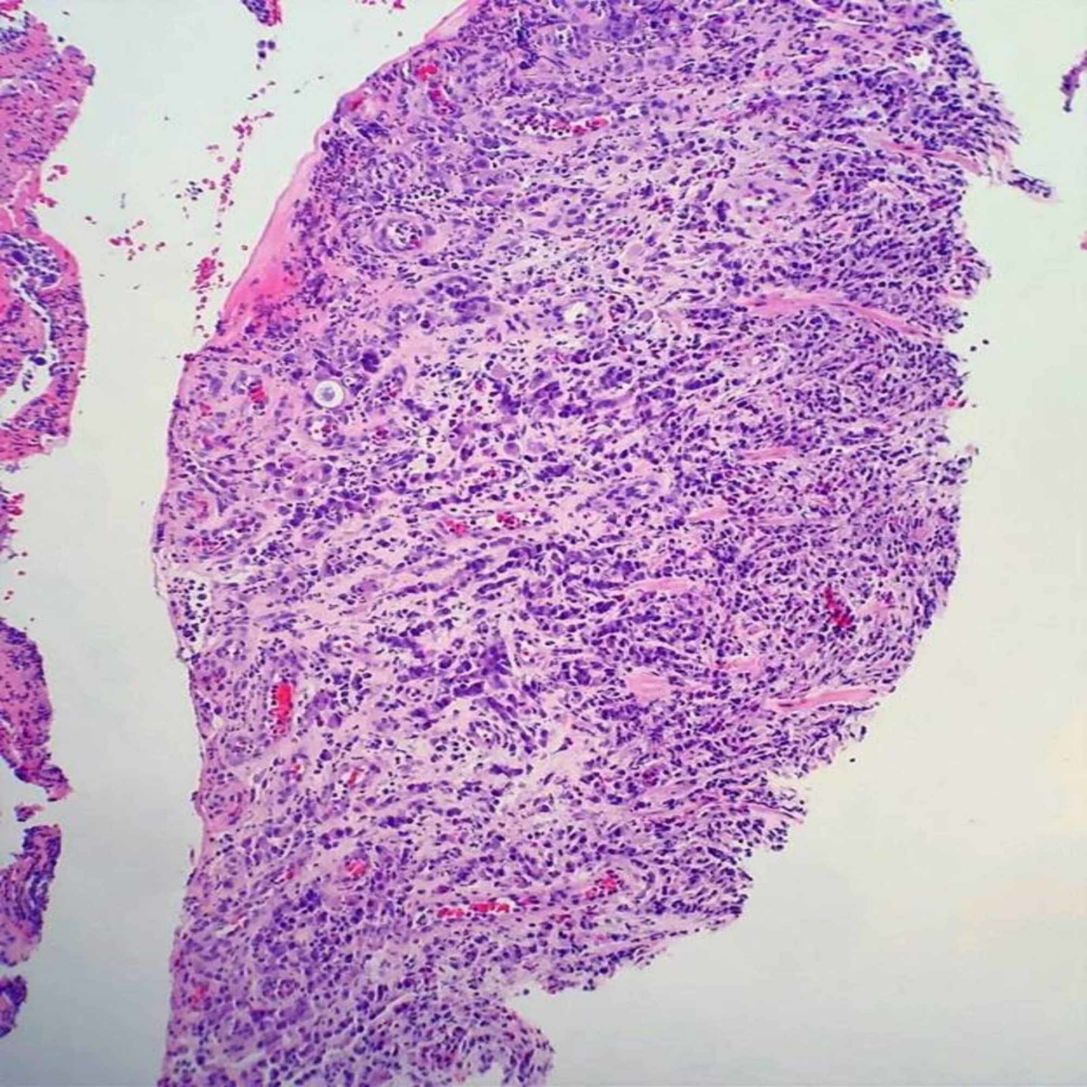
Stomach biopsy - ulcerated mucosa showing infiltrating gastric carcinoma

**Figure 5:**
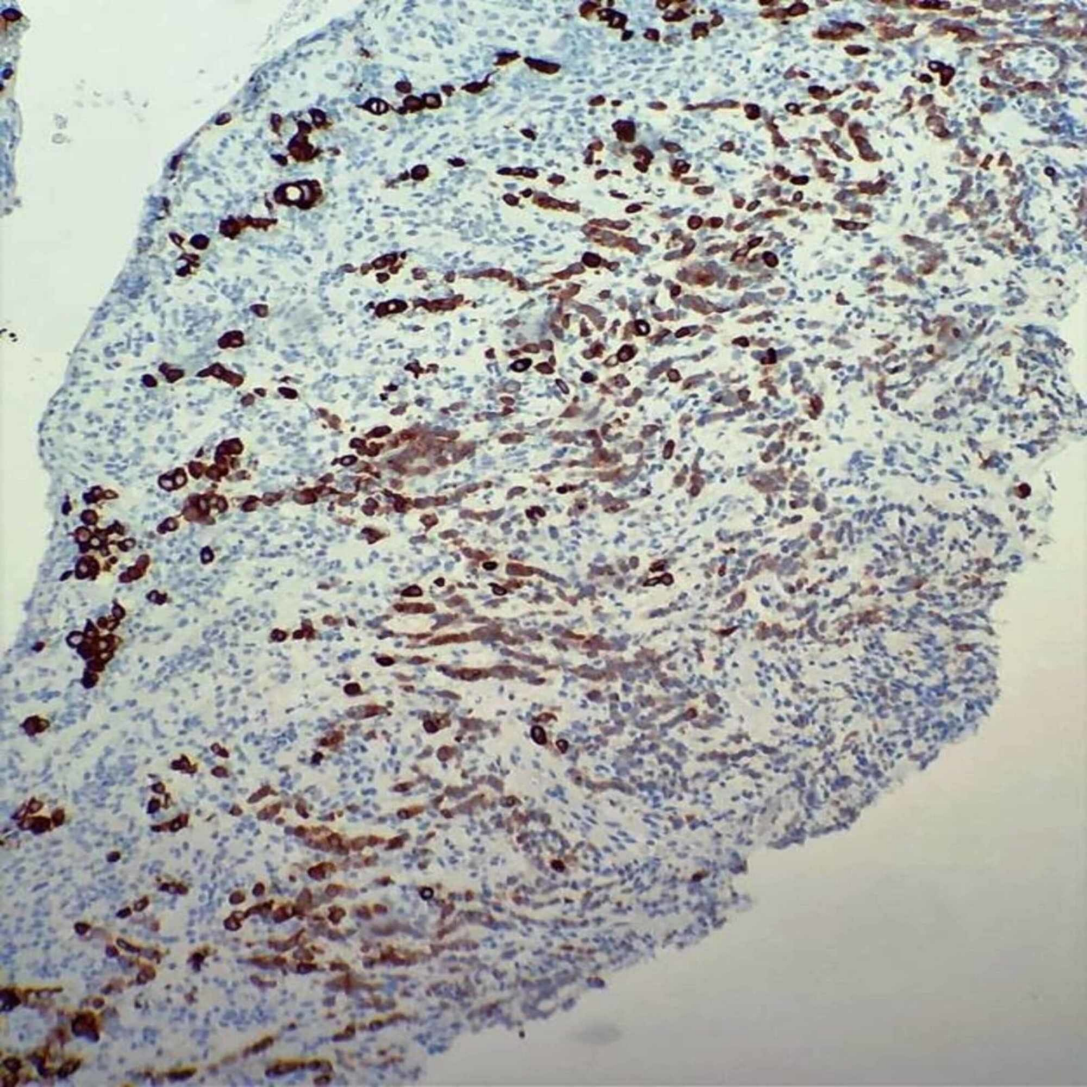
Stomach biopsy - pancytokeratin immunohistochemical stain confirming epithelial origin of infiltrating neoplastic cells

**Figure 6:**
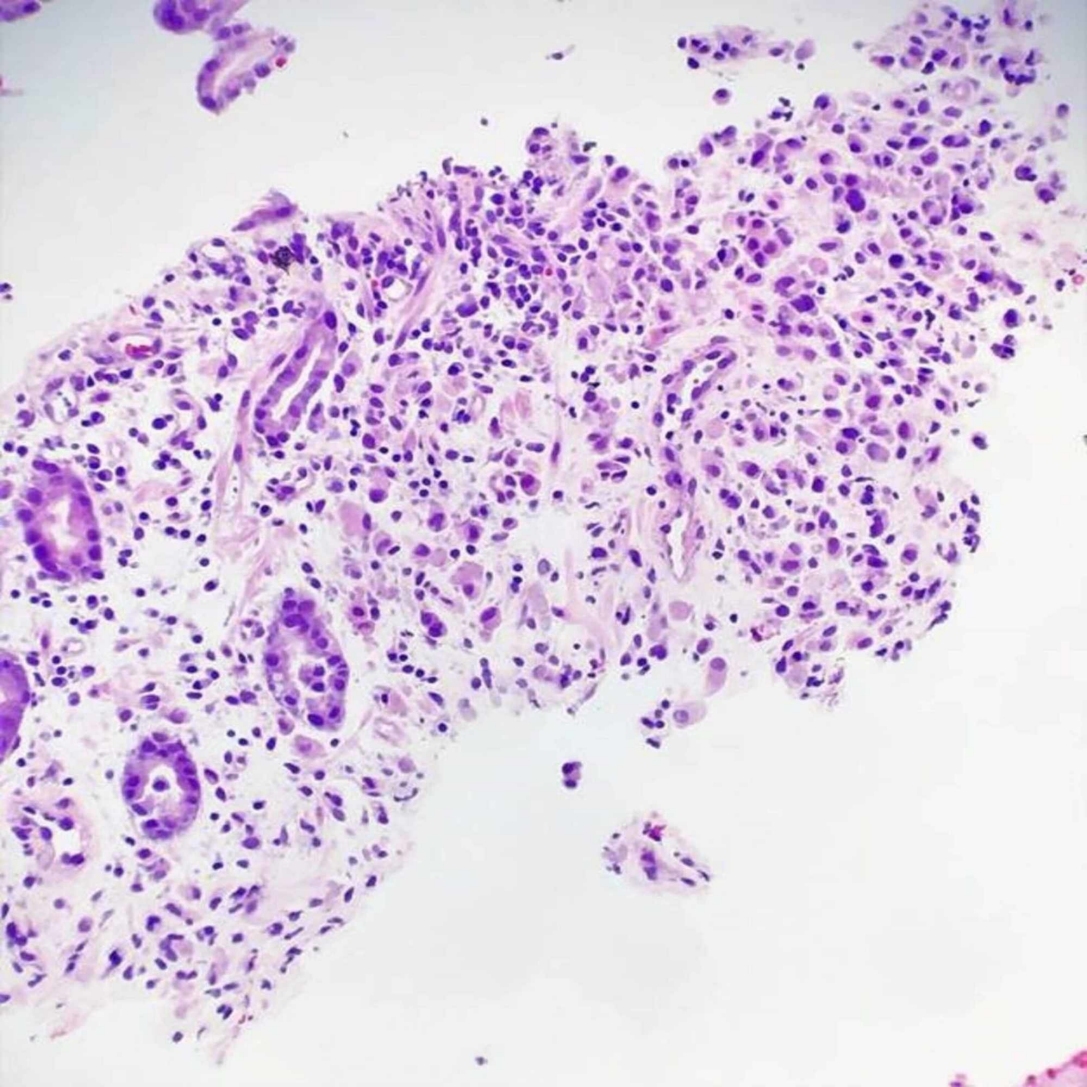
Colon biopsy - lamina propria subtly infiltrated by atypical epithelial cells with morphology similar to the gastric carcinoma

**Figure 7:**
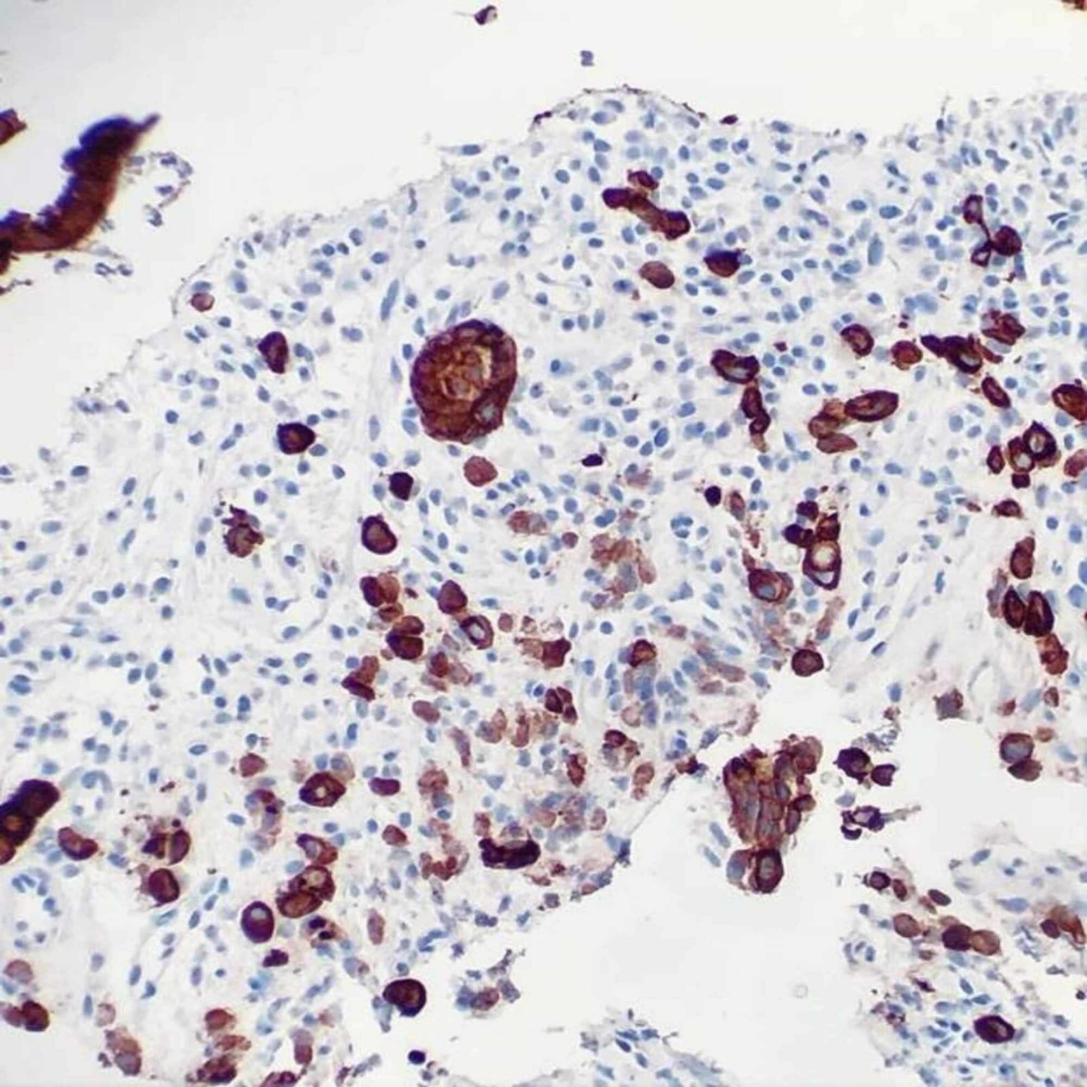
Colon biopsy - pancytokeratin immunohistochemical stain highlighting infiltrative epithelial cells, confirming metastatic carcinoma

A diagnostic paracentesis was performed due to his large volume ascites with drainage of three liters of yellow. Fluid was sent to pathology for cytology and immunohistochemical tests. Peritoneal fluid revealed presence of malignant cells compatible with peritoneal involvement of gastric adenocarcinoma. He underwent colonoscopy and an upper endoscopy with biopsies. Biopsies of the stomach showed invasive poorly differentiated adenocarcinoma, diffuse type, with associated ulceration; immune histochemical stain confirmed epithelial origin of infiltrating neoplastic cells (Figures 4-5). The colonic biopsy revealed lamina propria subtly infiltrated by atypical epithelial cells with morphology similar to the gastric carcinoma and immune histochemical stain highlighting infiltrative epithelial cells, confirming metastatic carcinoma (Figures 6-7). The diagnosis of gastric carcinoma with colonic and peritoneal metastases was made. The histopathological evidence and further cancer staging with magnetic resonance imaging (MRI) head and neck and positron emission tomography (PET) scan revealed that the gastric carcinoma is confined to the abdomen. The evidence of the metastatic disease on peritoneal fluid analysis, colonoscopy, and upper gastro endoscopy findings ruled out surgical intervention. A decision was made to initiate chemotherapy after a multidisciplinary discussion with the hospitalist team, Oncology, and General surgery. The patient agreed to initiate chemotherapy. He was started with 5‐fluorouracil, leucovorin, oxaliplatin (FOLFOX) chemotherapy regimen following the week of discharge. We also switched the anticoagulant to apixaban, a novel oral anticoagulant (NOAC). On short-term follow up, the patient received a total of six cycles of chemotherapy. He is tolerating chemotherapy well to date.

## Discussion

Abnormal umbilical pathologies are rare and can be classified as benign and malignant. Benign conditions are umbilical hernia, granuloma, abscess, eczema, and mycosis. Malignant tumors can be either primary umbilical origin or metastatic [4]. On physical examination, its appearance is often misleading because skin overlying the lesion can be normal or erythematous [5]. Review of literature showed 60% of the nodules were benign, and as a result, the diagnosis may be delayed [6]. The umbilical lesion can be easily missed due to its insidious presentation. Patients may attribute it to causes such as an insect bite, similar to our patient. This goes in conjunction with the insidious onset of an underlying gastric adenocarcinoma which usually presents as benign dyspepsia without alarm symptoms, even during advanced and inoperable states [7].

When an umbilical nodule is found, it is necessary to make an accurate histological diagnosis between the primary and metastatic lesion and the easiest method is to do a fine needle aspiration cytology and core biopsy. This is a feasible and inexpensive diagnostic tool [8]. Biopsy was recommended but considering the presentation of diarrhea and weight loss and CT findings of pulmonary thromboembolism, gastric varices, ascites, and bowel wall thickening, we had enough evidence for intra-abdominal or gastric visceral malignancy. His findings prompted us to consult gastroenterology who ultimately opted for endoscopic intervention. As a result, the patient underwent esophagogastroduodenoscopy and colonoscopy including biopsies at various sites. He was subsequently started on treatment per oncology services. 

The identification of cutaneous metastases heralds a poor prognosis [9]. Upon initial encounter of the patient, the clinical and radiologic evidence of pulmonary thromboembolism needed acute stabilization of the patient hemodynamically. Physical examination finding of SMJN and unprovoked venous thromboembolisms on diagnostic imaging studies were substantial clues. The most common primary site is a gastrointestinal tract neoplasm and upper gastrointestinal endoscopy and colonoscopy with a tissue diagnosis is a reasonable investigation. The mean life expectancy is 2 - 11 months without treatment; aggressive treatment with chemo radiation and/or surgical debulking has shown a mean survival of 17.6 - 21 months [10]. The patient, in this case, received his diagnosis within 48 hours of admission and was started on chemotherapy in five days of admission and with timely follow-up.

## Conclusions

Our patient presented with shortness of breath and leg swelling. Imaging studies revealed deep vein thrombosis and bilateral pulmonary embolism. A palpable umbilical nodule consistent with the appearance of SMJN raised the possibility of an underlying gastrointestinal malignancy. Endoscopic findings revealed gastric adenocarcinoma with colonic metastases. The patient was started on chemotherapy with FOLFOX for disseminated gastric malignancy. The presence of SMJN is a rare and a poor prognostic sign of a disseminated malignancy. Presentation of comorbidities with visceral advanced malignancy and pulmonary thromboembolism indicates poor prognosis; however, a timely diagnosis and urge of initiation of treatment will facilitate treatment for improved quality of life.
